# Evaluation of Serodiagnostic Assays for *Mycobacterium bovis* Infection in Elk, White-Tailed Deer, and Reindeer in the United States

**DOI:** 10.1155/2012/563293

**Published:** 2012-06-25

**Authors:** Jeffrey T. Nelson, Kathleen A. Orloski, Audra L. Lloyd, Mark Camacho, Mark A. Schoenbaum, Suelee Robbe-Austerman, Bruce V. Thomsen, S. Mark Hall

**Affiliations:** ^1^National Veterinary Services Laboratories, Veterinary Services (VS), Animal and Plant Health Inspection Service (APHIS), United States Department of Agriculture (USDA), Ames, IA 50010, USA; ^2^VS, APHIS, USDA, Fort Collins, CO 80526-8117, USA; ^3^VS, APHIS, USDA, Raleigh, NC 27606, USA

## Abstract

In 2011, the United States Department of Agriculture conducted a project in which elk (*Cervus elaphus* spp.), white-tailed deer (WTD) (*Odocoileus virginianus*), and reindeer (*Rangifer tarandus*) were evaluated by the single cervical tuberculin test (SCT), comparative cervical tuberculin test (CCT), and serologic tests. The rapid antibody detection tests evaluated were the CervidTB Stat-Pak (Stat-Pak), and the Dual Path Platform VetTB (DPP). Blood was collected from presumably uninfected animals prior to tuberculin injection for the SCT. A total of 1,783 animals were enrolled in the project. Of these, 1,752 (98.3%) were classified as presumably uninfected, based on originating from a captive cervid herd with no history of exposure to TB. Stat-Pak specificity estimates were 92.4% in reindeer, 96.7% in WTD, and 98.3% in elk and were not significantly different from SCT specificity estimates. Using the DPP in series on Stat-Pak antibody-positive samples improved specificity in the three species. Thirty one animals were classified as confirmed infected, based on necropsy and laboratory results, and 27/31 were antibody positive on Stat-Pak for an estimated sensitivity of 87.1%. The study findings indicate that rapid serologic tests used in series are comparable to the SCT and CCT and may have a greater ability to detect TB-infected cervids.

## 1. Introduction

*Mycobacterium bovis *has been detected sporadically in captive cervids in the United States and is enzootic in free-ranging white-tailed deer (WTD) (*Odocoileus virginianus*) in a small geographic area of Michigan [1, 2]. A multistate outbreak of *M. bovis* involving 37 captive cervid herds occurred in the United States during 1990–1999 [3]. The cervid species involved in this outbreak included elk (*Cervus elaphus *spp.), red deer (*Cervus elaphus*), fallow deer (*Dama dama*), and sika deer (*Cervus nippon*). *M. bovis* was detected again, beginning in 2009, in elk, red deer, and fallow deer in 4 herds located in Nebraska and Indiana [4, 5].

Testing captive cervids for *M. bovis* in the United States is conducted as part of an official disease eradication program administered by the United States Department of Agriculture (USDA). The single cervical tuberculin skin test (SCT) was first evaluated for use in elk in the United States in 1991 [6]. Captive cervids were not routinely tested for bovine tuberculosis (TB) until these species were officially brought into the federal tuberculosis program in 1994 [7]. Currently, testing for TB in cervids is performed using tuberculin skin tests. The SCT is the initial test and consists of the intradermal administration of bovine-purified protein derivative (PPD) tuberculin in the midcervical region [8]. Animals with any detectable responses are tested by the comparative cervical tuberculin skin test (CCT), in which balanced bovine and avian PPD tuberculins are injected, the pre- and postinjection skin thickness is measured, and the measurements are plotted on a graph [8]. Areas of the graph classify the animal as negative, suspect, or reactor. In cervids, the CCT must be administered within 10 days or after 90 days of the SCT tuberculin injection.

Limited and at times conflicting information is available regarding the performance of TB skin testing in captive cervid species. A comprehensive evaluation of cervid TB testing estimated the individual animal specificity of the SCT and CCT used in series to be 87.1 and 90.4% for deer and elk, respectively [9]. Individual animal sensitivity of the SCT and CCT in series could not be estimated; however, 14 elk from 12 infected herds detected between 1991 and 1996 were CCT test negative and subsequently confirmed to be infected with *M. bovis*. More recently, 25/28 confirmed infected Nebraska elk had a false negative result on the SCT [4]. The estimated specificity of the CCT in reindeer (*Rangifer tarandus*) is significantly different than nonreindeer cervids, with reindeer approximately four times more likely to test false positive on the CCT [10]. Eleven experimentally infected reindeer tested positive by the CCT at three and eight months after infection [11]; however, estimates of sensitivity of CCT in naturally infected reindeer are not possible as TB is extremely rare in this species.

In addition to concerns regarding TB skin test performance, animal handling challenges resulting in animal morbidity and mortality are not uncommon. With TB skin testing, captive cervids may be required to be captured and restrained for testing up to four different times depending on test results. Serologic testing offers the advantage over skin testing of reduced animal handling, with a reduction in the associated morbidity and mortality. An additional advantage is eliminating the subjectivity of interpreting the skin response at the tuberculin injection site. For these reasons, there is potential for improved surveillance in captive cervids using a serologic test with adequate performance.

In 2009, the CervidTB Stat-Pak lateral flow test (Stat-Pak, Chembio Diagnostic Systems, Inc., Medford, New York) was licensed for use in elk and red deer. The Stat-Pak is a rapid point-of-care test that utilizes single-directional lateral-flow serological antibody detection technology and a cocktail of recombinant *M. bovis *antigens [12]. In addition to the Stat-Pak, Chembio has two additional antibody detection assays for TB, the multiantigen print immunoassay (MAPIA) and the recently developed dual path platform VetTB (DPP) assay [13]. The MAPIA is performed in the laboratory as a confirmatory testing method for samples with antibody-positive results on the Stat-Pak. The DPP is a new-generation point-of-care test format that offers improved specificity compared to the Stat-Pak assay with similar sensitivity in red deer, elk, and fallow deer [4, 14].

The primary objective of this study was to evaluate the Stat-Pak as a primary test for use in the diagnosis of TB in captive and free-ranging elk, WTD, and reindeer. In addition, the DPP was evaluated as a followup test for Stat-Pak antibody-positive samples, and the two serologic test results were compared to TB skin test results.

## 2. Methods

Blood samples from two sources were tested as part of the project: blood collected prospectively during triennial herd TB accreditation testing and banked serum. The species evaluated included elk, WTD, and reindeer. Banked serum was from the TB serum bank located at the National Veterinary Services Laboratories (NVSL) in Ames, Iowa.

Banked serum samples were derived from animals sampled during 2008–2010 and included samples from elk, WTD, and reindeer with corresponding SCT results. Most banked samples were from captive cervid herds with no history of exposure to TB with the exception of one infected elk herd that was detected and depopulated in 2009. Some samples reported by Waters et al. [4] were retested for the sensitivity portion of this project. Prospectively enrolled animals were sampled during calendar year 2011. Captive cervid producers were recruited to enroll animals in the project through producer organizations. In addition, free-ranging cervids being TB tested (such as for wildlife restoration projects) were enrolled. The animals sampled prospectively came from 51 separate premises, including 16 for elk, 3 for reindeer, and 32 for WTD herds.

Accredited or regulatory veterinarians collected blood samples. Blood was collected into 10 mL serum collection tubes or serum separator tubes on the day of tuberculin injection for the SCT. An exception to these methods occurred with the infected elk herd; SCT testing was conducted three months prior to the animals being euthanized and necropsied, and blood collection was done immediately after postmortem at necropsy. Blood samples were centrifuged, separated, and shipped to NVSL. Alternatively, unseparated blood was cooled and shipped overnight to NVSL, where the serum was separated. Copies of the completed SCT results were submitted with blood or serum [8].

Prospectively sampled animals that were antibody positive on the Stat-Pak were further evaluated by the CCT and were later necropsied. However, for some cases, the owners chose not to have their animals CCT tested and euthanized. Animals that responded to the SCT test were administered a CCT according to current USDA TB program requirements, regardless of Stat-Pak results (Table 1) [15]. The USDA TB program requires that CCT-positive animals are euthanized and necropsied, with tissue samples taken for histopathology and bacteriologic culture; CCT-negative animals are released from quarantine.

Tissue samples from necropsied animals were divided, and the two sections were submitted separately in 10% buffered formalin and a saturated sodium borate solution for histopathology and bacteriologic culture, respectively. Head and thoracic cavity lymph nodes and any lesioned tissues were submitted from necropsied animals. No followup was conducted on animals testing negative on the Stat-Pak, although some reindeer sampled in 2011 were subsequently slaughtered and inspected. Histopathology and bacteriologic culture were conducted at the NVSL.

Tissues submitted in 10% buffered formalin were initially evaluated for lesions and the presence of acid-fast bacilli by histopathology. Tissues were diagnosed as compatible with mycobacteriosis when granulomas were identified that contained acid-fast bacilli. Tissues for culture were prepared as previously described [16]. All submitted tissues were cultured using at least one liquid media system (BACTEC 460 or MGIT 960) and 2 tubes of a modified Middlebrook 7H11 supplemented with calf serum, hemolyzed blood, pyruvate, and malachite green.

The Stat-Pak was performed at NVSL per the manufacturer's instructions. Serologic and TB testing in the infected elk herd have been previously described [4]; however, in this study, serum samples from this herd were tested by the Stat-Pak at NVSL. All sera with an antibody-positive result on the Stat-Pak were further tested via the MAPIA and DPP at Chembio, Inc., in Medford, New York as described previously [12–14].

Animals that are necropsied as a result of official TB testing or antibody-positive Stat-Pak results and have microscopic lesions that are compatible for mycobacteriosis, and/or *M. bovis *is isolated from tissue, were classified as TB infected. Animals were classified as presumably uninfected if they were from herds with no history of exposure to TB and resided in states declared free of TB in cattle. Animals that did not meet the case definition for confirmed TB infected but were from herds with infected animals were classified as exposed and not included in the analysis.

The sample size estimates were derived from Greiner and Gardner [17]. Sensitivity was defined as test positives/test positives + test negatives using the sample of animals that met the case definition for confirmed TB infected. Specificity was defined as test negatives/test negatives + test positives for animals meeting the case definition for presumably uninfected. Ninety five % confidence intervals for proportions were calculated using the *F* statistic function in Excel (function = F.INV.RT, Microsoft, 2010). Tests of statistical significance were performed using the chi-square, Fischer exact and mid-p exact, and were calculated online using Open Epi (http://www.openepi.com/OE2.3/Menu/OpenEpiMenu. htm). Results were considered statistically significant for *P* values <  0.05.

## 3. Results

A total of 1,783 animals were tested by the Stat-Pak, including 873 elk, 725 WTD, and 185 reindeer. Of these, 1,752 (98.2%) were classified as presumably uninfected, and 31 animals (elk from one affected herd) were classified as confirmed TB infected.

### 3.1. Presumably Uninfected Animals

Of the samples from presumably uninfected animals, 52/1752 (3.0%) were antibody positive by the Stat-Pak assay, including 14/842 elk (1.7%), 24/725 WTD (3.3%), and 14/185 reindeer (7.6%) (Table 2). Twenty six of 853 (3.1%) samples from banked serum and 26/899 (2.9%) samples collected prospectively were antibody positive by the Stat-Pak assay. Of the 26 antibody-positive animals sampled prospectively, 9 were elk, 13 were WTD, and 4 were reindeer. Of these, a necropsy was completed on 7 elk, 4 WTD, and 1 reindeer with no evidence of *M. bovis* infection. For the remaining 14 animals, the owners declined to have the animals euthanized for necropsy. One reindeer and one elk had a histopathologic diagnosis of microgranuloma. Two elk had a histopathologic diagnosis of lymphoid hyperplasia. *Mycobacterium intracellulare* and an unidentified atypical mycobacterium were isolated from the elk with lymphoid hyperplasia; *M. intracellulare* was isolated from an elk with no significant findings on histology.

The estimated specificity of the Stat-Pak by species is 98.3% in elk, 96.7% in WTD, and 92.4% in reindeer in this study (Table 2). Specificity was significantly different for the three species (*P* = 0.00008, uncorrected chi-square). SCT data from USDA TB Program official testing during FY 2009 provided an estimated specificity of 98.5% for elk, 97.4% for WTD, and 82.7% for reindeer, from a sample of over 6,700 presumably uninfected animals (Table 3). For elk and WTD, the estimated specificity of the Stat-Pak compared to the FY 2009 SCT was not significantly different (*P* >0.05, mid-p exact). In reindeer, significantly fewer animals were antibody positive by the Stat-Pak compared to responders on the SCT (*P* = 0.024, mid-p exact). The USDA TB Program testing requires that SCT responders be administered the CCT test. During routine USDA TB Program testing during FY 2009-2010, 5/123 (4.1%) elk were positive on the CCT, and 118 were negative. For WTD, 6/119 (5.0%) animals were positive on the CCT, and 113 animals were classified negative. In reindeer, 19/19 CCT tested animals were negative. The specificity of the SCT and CCT tests in series could not be calculated.

USDA has reported the estimated specificity of the SCT and CCT used in series to be 87.1% (95% CI 84.5–89.4) in deer and 90.4% (95% CI 87.4–92.9) in elk and red deer [9]. This was significantly lower than the estimated specificity of the Stat-Pak and DPP used in series for WTD and elk (*P* < 0.0001, mid-p exact). A second study by Norden et al. [18] reported higher specificity estimates for the SCT and CCT in series of 98.3% (95% CI 96.0–99.5) in deer and 99.5% (95% CI 95.4–98.6) in elk and red deer, in which animals classified as “suspects” (weak positives) on the CCT were reclassified as negative. Comparing the 1997 SCT and CCT in series results to the current study, the specificity estimates for the Stat-Pak and DPP in series were not significantly different for WTD but were different for elk (*P* = 0.000036, mid-p exact), with the Stat-Pak/DPP combination having a higher specificity than the SCT/CCT. Test performance for reindeer was also reported in Norden et al. [18], where 29/29 reindeer were negative when tested in series by the SCT and CCT, compared to 182/185 Stat-Pak/DPP test negatives in the present study.

Nearly 62% of the animals evaluated were female (993/1608). The highest percentage of female animals occurred in reindeer (150/185 female) (Table 2). Gender was not reported for 144 animals. When stratified by species, test performance was not significantly different between females and males in this study (*P* = 0.575, elk; *P* = 0.969, reindeer; *P* = 0.282, WTD; mid-p exact).

Samples collected during 2011 were used to compare the performance of the Stat-Pak with the SCT (Table 4). A total of 879 animals had results for both the SCT and the Stat-Pak. For elk, 7/344 (2.0%) animals were SCT positive, while 9/344 (2.6%) were antibody positive on the Stat-Pak. For WTD, 13/494 (2.6%) and 13/494 (2.6%) animals were SCT positive and Stat-Pak antibody positive, respectively. SCT results were available for 41 reindeer. Of these, 9/41 (22.0%) and 4/41 (9.8%) were SCT positive and Stat-Pak antibody positive, respectively. Three reindeer and two elk were SCT positive and antibody positive on the Stat-Pak. In all other cases, the Stat-Pak antibody-positive animals were different individuals than the SCT-positive animals. CCT tests were administered to 11 Stat-Pak antibody-positive animals. Three elk, four reindeer, and two WTD were CCT negative. Two WTD were classified as positive on the CCT.

Fifty two samples antibody positive on Stat-Pak were tested by the DPP and MAPIA (Table 5). Both assays use an expanded panel of antigens in addition to the antigens included in the Stat-Pak, to improve specificity. The largest improvement in specificity was observed in elk, where 13/14 samples (each antibody positive on Stat-Pak) were negative by the MAPIA, and only one sample was positive (Table 5). In WTD, 13/24 (54.2%) antibody positive on the Stat-Pak were negative on the MAPIA. In reindeer, 11/14 (78.6%) antibody positive on the Stat-Pak were negative on the MAPIA. The DPP had similar results to the MAPIA. The increase in specificity estimates from the Stat-Pak only to the combination of the Stat-Pak and DPP tests used in series were 98.3% to 100.0% (842/842, 95% confidence interval (CI) 99.6–100.0) in elk; 96.7% to 99.3% (720/725, 95% CI 98.4–99.8) in WTD; 92.4% to 98.4% (182/185, 95% CI 95.3–99.7) in reindeer.

### 3.2. Serology Test Performance in a TB-Affected Elk Herd

A total of 34 animals from a known TB-affected Nebraska elk herd were evaluated for the project. Thirty one animals met the case definition for confirmed TB infected and had a serum sample available for inclusion in the project. Of these, 27 (87.1%) were antibody-positive by the Stat-Pak, and followup testing resulted in 26 and 27 antibody positive samples by the MAPIA and DPP, respectively. Twenty of the 31 confirmed infected animals were SCT tested with negative results three months prior to being necropsied. Among these 20 SCT negative animals, 18 (90.0%) were Stat-Pak antibody positive, and 2 were negative. Three animals from the infected elk herd did not meet the definition for confirmed TB infection; these animals were considered exposed to TB and were excluded from the analysis. Lesioned tissues from these 3 animals were compatible for mycobacteriosis by histopathology, but *M. bovis* was not isolated. For these 3 animals, two serum samples were negative, and one was antibody positive on the Stat-Pak and the DPP.

## 4. Discussion

The estimated specificity of the Stat-Pak from this study was not significantly different from the SCT in elk and WTD and was higher in reindeer compared to the SCT. This finding is similar to a study in which significant differences in SCT test performance between cervid species were found [9]. A substantial improvement in specificity was observed when the Stat-Pak was used in series with either the DPP or MAPIA. The increase in specificity varied by species with the largest improvement occurring in elk and the lowest in WTD. In this study, the specificity estimates for the Stat-Pak and DPP used in series for WTD and elk are significantly higher than estimates of the SCT and CCT used in series in deer and elk [9]. However, the 1996 estimate for deer may not accurately reflect test performance in WTD, as multiple species were included as deer and could have included reindeer, roe, fallow, and other species, in addition to WTD.

In this study, the specificity estimates for the Stat-Pak and DPP were similar to other reports. Waters et al. [4] reported an estimated specificity of the DPP of 98%, while in New Zealand red deer, a species closely related to elk, the estimated specificity was 98.3% [14]. A previous study of the Stat-Pak found an estimated specificity in WTD of 98.9% [12].

While this project was primarily focused on specificity because of the limited number of samples from infected cervids, sensitivity is also an important consideration. In this study, the sensitivity of the Stat-Pak was 87.1%; however, this estimate was determined from a relatively small number of animals in a single herd. Buddle et al. [14] reported a sensitivity for the Stat-Pak and DPP of 75% each (not used in series) in naturally infected red deer. Waters et al. [4] noted that the SCT performed poorly in detecting TB, as only 3/28 confirmed infected elk were SCT positive. The failure of the skin test to detect infection in elk has been documented previously; the CCT was negative in 14 elk that were subsequently confirmed to be infected with *M. bovis* [9]. In the present study, three months elapsed between the time the SCT was administered and blood samples were collected for serologic testing. It is possible that the SCT-negative results occurred in animals that were not yet infected; however, at necropsy many animals in the herd had advanced clinical disease [4], making this explanation less likely. However, Norden et al. [9] described that several of 14 confirmed infected elk that were initially CCT negative were CCT positive when tested again at a later date.

Samples from naturally infected WTD and reindeer were not available for this project; however, published studies have reported Stat-Pak sensitivity ranging between 55% and 67% in naturally infected, free-ranging WTD, and 79% in experimentally infected animals [12, 19]. The estimated sensitivity of the CCT administered as the only skin test in experimentally infected WTD was 97% [20]. An evaluation of 11 experimentally infected reindeer found that *M. bovis*-specific antibody was found as early as four weeks after infection [11]. No naturally infected reindeer have been detected in the US.

Animals that were positive on the Stat-Pak were different than the animals that responded to the SCT, with only a few exceptions. One possible explanation for this finding may be the basic differences between these tests, with one measuring humoral, and the other, cell-mediated immunity. Additionally, the serologic tests use specific antigens, while PPD tuberculins are a complex mixture of many cross-reactive antigens. This finding has important implications for choosing a supplemental test. The CCT may not be an appropriate supplemental test to use in series with the Stat-Pak, because some of the sensitivity gained using the serologic test could be lost using the skin test. Alternatively, measuring different aspects of immunity may be more robust at detecting infection than two tests that measure the same immune response components. For example, the humoral immune response may not be subject to anergy, which is known to cause false-negative skin test results.

The primary study limitation was that animals testing negative on the Stat-Pak were not necropsied to confirm that they were not infected with *M. bovis*, with the exception of slaughter inspection conducted in some reindeer sampled prospectively. Given the recent findings of *M. bovis* in several US captive cervid herds, there is a small risk that infected animals classified as presumably uninfected were included in the study. Additionally, the samples included in this study were a convenience sample of animals being TB tested for routine purposes and subject to producers volunteering to participate. For example, a number of producers that originally agreed to participate in the study declined after learning of the requirement to euthanize animals testing positive on the Stat-Pak and a maximum indemnity payment of $3,000. Only 12/26 prospectively sampled antibody-positive animals were necropsied because producers declined to have their animals euthanized and necropsied. To avoid these limitations in the future, studies would need adequate funding and industry cooperation to more thoroughly evaluate the disease status for both test-negative and -positive animals.

In addition to the potentially higher diagnostic accuracy of antibody detection methods evaluated in the present study, point-of-care serologic testing by simple and rapid assays offers the advantage over skin testing of reduced animal handling and the associated morbidity and mortality and eliminates the subjectivity associated with evaluating the tuberculin injection site for a response. It is anticipated that growing acceptance of this approach by producers will result in gradually expanded TB testing to further improve overall TB surveillance in captive and free-ranging cervid populations.

## Figures and Tables

**Table 1:** Decision algorithm for supplemental testing.

	Single cervical tuberculin (SCT) test result	
Stat-Pak result	SCT negative	SCT positive
Negative	No additional testing	Follow USDA TB program regulations [15]
Positive	MAPIA and DPP, purchase with indemnity	MAPIA and DPP, follow USDA TB program regulations

**Table 2:** Stat-Pak results in presumably uninfected animals.

Species	Sex	Stat-Pak Negative	Stat-Pak Antibody Positive	Total Tested	Percent Positive	95% Confidence Interval
Elk	Female	428	7	435	1.6%	0.6–3.3%
	Male	338	3	341	0.9%	0.2–2.5%
	Not known	62	4	66	6.1%	—
*Elk Total*		828	14	842	1.7%	0.9–2.8%
Reindeer	Female	138	12	150	8.0%	4.22–13.6%
	Male	33	2	35	5.7%	0.7–19.2%
*Reindeer Total*		171	14	185	7.6%	4.2–12.4%
WTD*	Female	391	17	408	4.2%	2.4–6.6%
	Male	233	6	239	2.5%	0.9–5.4%
	Not known	77	1	78	1.3%	—
*WTD Total*		701	24	725	3.3%	2.1–4.9%
Grand Total		1700	52	1752	3.0%	2.2–3.9%

*WTD = white-tailed deer.

**Table 3:** Single cervical tuberculin test response fraction in elk, WTD and reindeer in federal fiscal year (FY) 2009.

Species	Total Tested	Responders	Percent Responders	95% Confidence Interval
Elk	3,223	47	1.5%	1.1–1.9
WTD*	3,421	89	2.6%	2.1–3.2
Reindeer	81	14	17.3%	9.8–27.30

Total	6,725	150	2.2%	1.9–2.6

*WTD = white-tailed deer.

**Table 4:** Comparison of single cervical tuberculin skin test (SCT) and the Stat-Pak, presumably uninfected animals, by species in 2011.

SCT Result*	Stat-Pak Negative	Stat-Pak Positive	Total
*Elk*			
Negative	330	7	337
Positive	5	2	7
Elk, total	335	9	344
*White-tailed deer (WTD)*			
Negative	468	13	481
Positive	13	0	13
WTD, total	481	13	494
*Reindeer*			
Negative	29	2	31
Positive	7	3	10
Reindeer, total	36	5	41

Total	852	27	879

*Includes only animals sampled prospectively during 2011.

**Table 5:** Results of multi-antigen print immunoassay (MAPIA) and dual-path platform VetTB (DPP) for Stat-Pak antibody positive samples.

	MAPIA Results*						
DPP Results	Elk		Reindeer		WTD		Total
	Neg	Pos	Neg	Pos	Neg	Pos	
Negative	13	1	10	1	13	6	44
Positive	0	0	0	3	0	5	8

Total	13	1	10	4	13	11	52

*Neg = negative, Pos = positive, WTD = white-tailed deer.
